# Supplementary material for: Polymer-dispersed liquid crystal elastomers as moldable shape-programmable material
Source: Nat Commun. 2023 Feb 10;14:764. doi: 10.1038/s41467-023-36426-y (PMC9918464; doi:10.1038/s41467-023-36426-y)
Supplement: Supplementary file 1 — Supplementary Information [file 41467_2023_36426_MOESM1_ESM.pdf]

Supplementary Information for Polymer-dispersed liquid crystal elastomers as moldable shape-programmable material

Matej Bobnar[1], Nikita Derets[1,2], Saide Umerova[1], Valentina Domenici[3], Nikola Novak[1], Marta Lavrič[1], George Cordoyiannis[1], Boštjan Zalar[1,4] and Andraž Rešetič[1]*

[1]*Jožef Stefan Institute, Solid State Physics Department, Jamova cesta 39, 1000, Ljubljana, Slovenia.*
[2]*On leave from: Ioffe Institute, Division of Physics of Dielectrics and Semiconductors, Politekhnicheskaya 26, 194021 St. Petersburg, Russia.*
[3]*Dipartimento di Chimica e Chimica Industriale, Università degli studi di Pisa, via Moruzzi 13, 56124 Pisa, Italy.*
[4]*Jožef Stefan International Postgraduate School, Jamova cesta 39, 1000, Ljubljana, Slovenia.*
E-mail: andraz.resetic@ijs.si

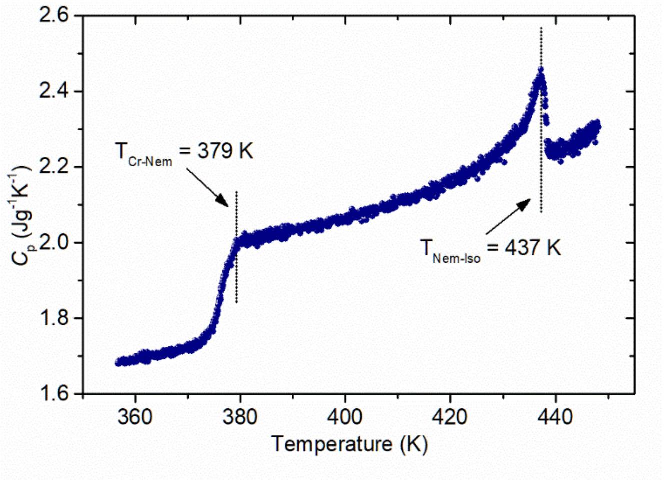

Supplementary Figure 1. The temperature profile of heat capacity $C_p(T)$ of the MC-LC upon cooling with a scanning rate of 0.3-0.5 K h^{-1}. The high-temperature peak corresponds to the isotropic (Iso) to nematic (Nem) phase transition, whereas the step at the lower temperatures is attributed to the transition from the nematic to glass state. The range of the weakly first order nematic to isotropic phase transition is ~ 14 K with a coexistence range of ~ 2.5 K. The $T_{Glass\text{-}Nem}$ phase transition temperature in the main text denotes the onset of the nematic to glass phase transition.

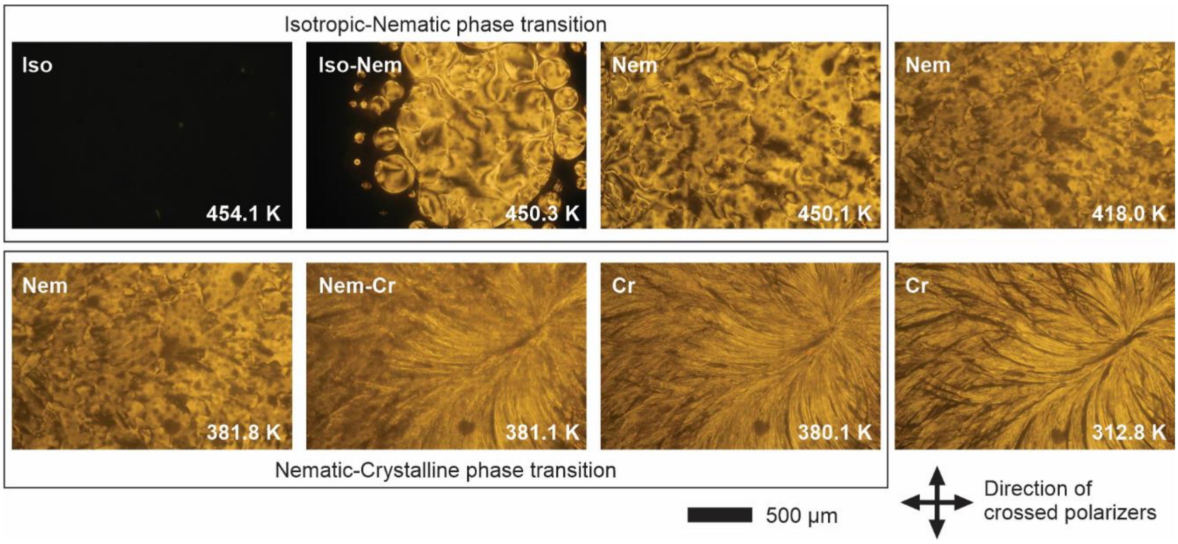

Supplementary Figure 2. Polarized microscopy pictures of phases found in MC-LCs. Images were taken upon cooling the sample from the isotropic to the glass phase. Note that the Nem-Iso phase transition temperature differs from the calorimetry measurements for temperatures above 400 K due to poor heat insulation of the heating cell.

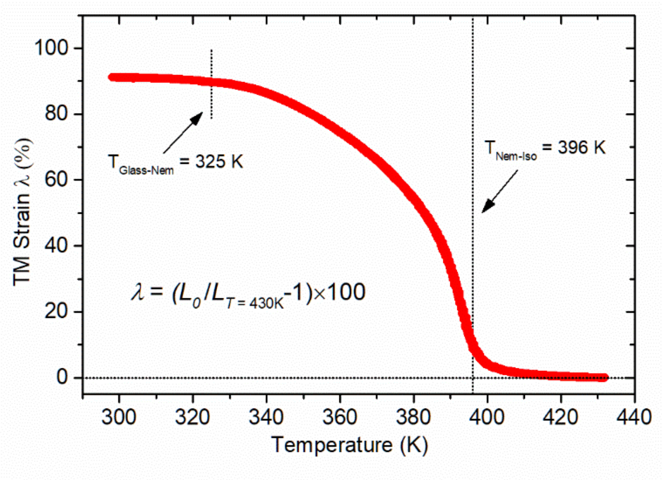

Supplementary Figure 3. TM measurements of a monodomain MC-LCE show a gradual contraction in strain of $\lambda_{\text{MC-LCE}} = 91$ % when heated from 300 K to 430 K, with two observable slope changes at the outset of $T_{\text{Glass-Nem}} \approx 325$ K and onset of $T_{\text{Nem-Iso}} \approx 396$ K, associated with the glassy to nematic and the nematic to isotropic phase transitions, respectively.

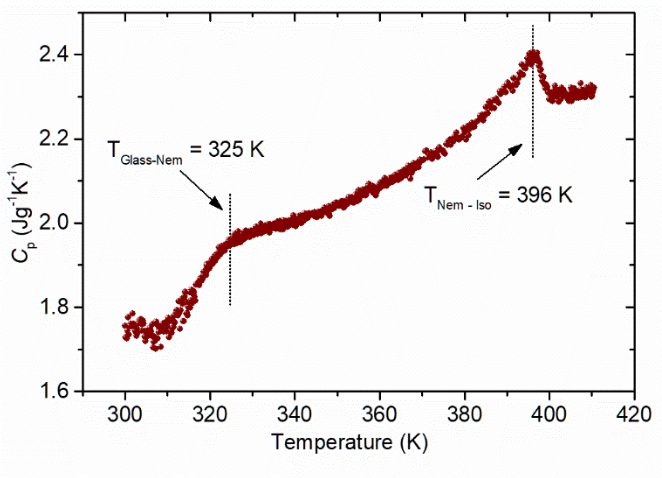

Supplementary Figure 4. The temperature profile of heat capacity $C_p(T)$ of a monodomain MC-LCE exhibits: (i) a weak first order transition from the nematic to the isotropic phase (Nem-Iso) at high temperatures and (ii) a step-like anomaly at lower temperatures denoting the onset of the glass phase transition. The measurement was taken upon cooling the specimen with a scanning rate of 0.8 K h^{-1}.

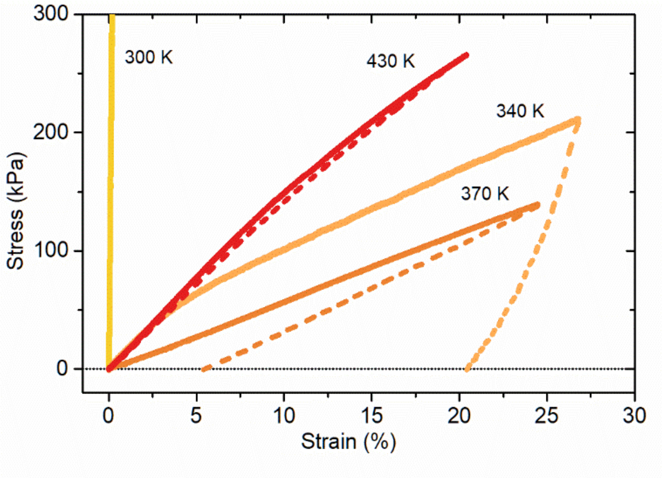

Supplementary Figure 5. Stress-strain measurements of a monodomain MC-LCE sample were performed at characteristic temperatures associated with the material's LC mesophases: crystalline phase (300 K), crystalline to nematic transition (340 K), nematic phase (370 K), and isotropic phase (430 K).

Programming fixation ratios					
Sample	MC-LCE	MC-LCE Polydomain	PDLCE	PDLCE High stress [b)]	PDLCE TM-active
R_{fix} Tensile [a)]	0.998 ± 0.001	0.995 ± 0.001	0.81 ± 0.02	0.80 ± 0.02	0.85 ± 0.01
R_{fix} Compressive [a)]	0.785 ± 0.002	/	0.80 ± 0.01	0.82 ± 0.01	0.80 ± 0.01

[a)] Applied stress: $\sigma_{tensile} \approx 15$ kPa, $\sigma_{compressive} \approx 80$ kPa; [b)] applied high stress: $\sigma_{tensile} \approx 50$ kPa, $\sigma_{compressive} \approx 400$ kPa.

Supplementary Table 1. The tensile and compressive stress programming strain fixation ratios, R_{fix}, for the investigated materials are listed in this table. While the strain fixation after compressive stress programming is significantly reduced for MC-LCEs, the values are comparable for PDLCEs. Furthermore, programming with higher stress does not seem to have any additional effect on R_{fix}. Error in R_{fix} is determined from three consecutive measurements.

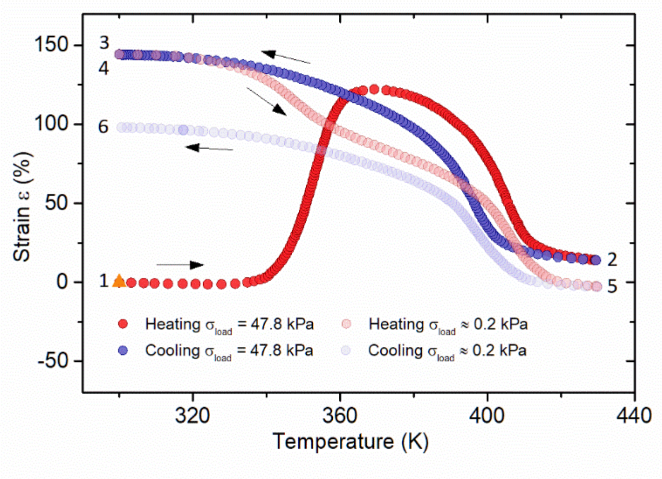

Supplementary Figure 6. Thermal-cycling of a polydomain MC-LCE sample with applied tensile stress shows especially high strain to applied mechanical stress. Large programmable strain leads to substantial relaxations during thermal reset, but the sample does not relax to the initial length of the system. Even a small applied load renders the material monodomain. The material returns entirely to its original length only if thermal-cycled while hanging freely or positioned horizontally on the surface.

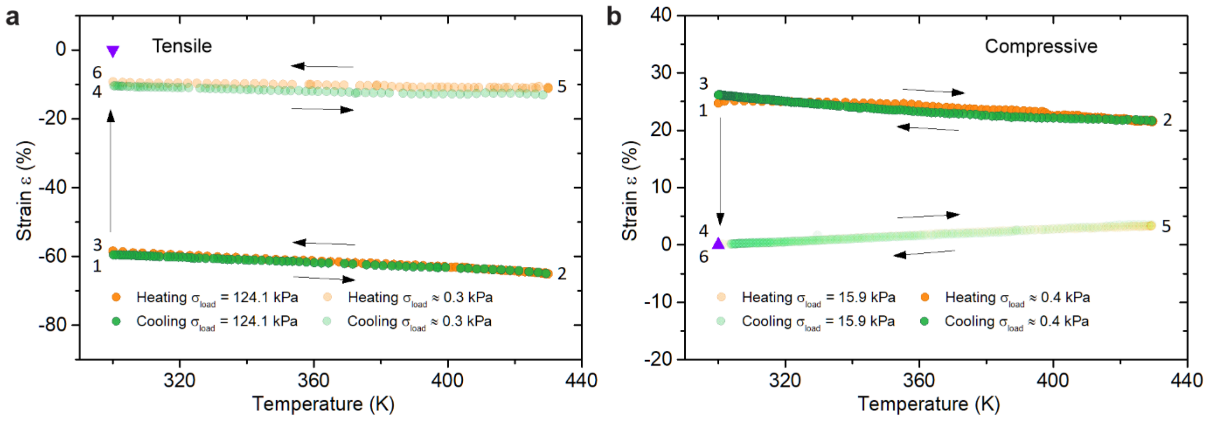

Supplementary Figure 7. Thermal-cycling of a PDMS sample with tensile (a) and compressive (b) programmable stress. The material is used as the elastic matrix in PDLCEs. In both cases, the PDMS material does not exhibit any shape-memory or hysteresis effects. Purple triangles denote the sample's length/thickness before the thermal-cycling.

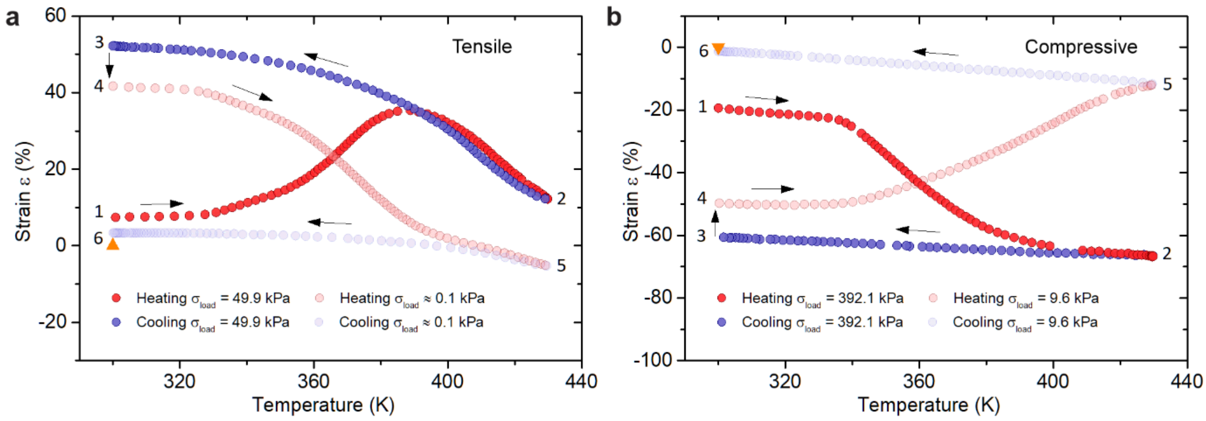

Supplementary Figure 8. High stress thermal-cycling of a PDLCE sample with tensile (a) and compressive (b) programmable stress. Compared to the initial measurements in Figure 3a, one can observe that the memorized strain relaxation temperatures have shifted to much higher temperatures, even well beyond the $T_{\text{Nem-Iso}}$ in the case of compressive programmable stress. Nevertheless, the curves maintain the same thermomechanical behaviour.

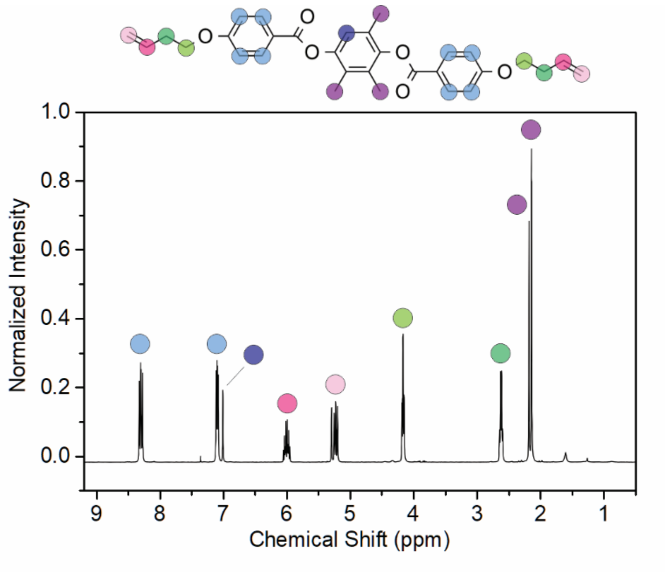

Supplementary Figure 9. ^{1}H-NMR spectra of synthesized divinylic mesogenic monomers (MC-LCs) in chloroform-d_1, used for the preparation of MC-LCE material.
