# Supplementary material for: Polymer-dispersed liquid crystal elastomers as moldable shape-programmable material
Source: Nat Commun. 2023 Feb 10;14:764. doi: 10.1038/s41467-023-36426-y (PMC9918464; doi:10.1038/s41467-023-36426-y)
Supplement: Supplementary file 3 — Description of Additional Supplementary Files [file 41467_2023_36426_MOESM3_ESM.pdf]

Description of Additional Supplementary Files

File Name: Supplementary Movie 1

Description: Bending shape programming

File Name: Supplementary Movie 2

Description: Torsion shape programming

File Name: Supplementary Movie 3

Description: Debossing and Embossing

File Name: Supplementary Movie 4

Description: Shape memory relaxation between solid 3D objects

File Name: Supplementary Movie 5

Description: In-plane morphing

File Name: Supplementary Movie 6

Description: Out-of-plane morphing

File Name: Supplementary Movie 7

Description: Solid object morphing
